# Theoretical study of induced selective N_2_ binding under an electric field in MOF-74: application for N_2_/CH_4_ separations†

**DOI:** 10.1039/d2ra04216a

**Published:** 2022-08-19

**Authors:** Honghui Kim, Jihan Kim

**Affiliations:** Department of Chemical and Biomolecular Engineering, Korea Advanced Institute of Science and Technology (KAIST) 291 Daehak-ro, Yuseong-gu Daejeon 34141 Republic of Korea jihankim@kaist.ac.kr

## Abstract

In this theoretical study, selective binding of dinitrogen to the coordinatively unsaturated metal site in M-MOF-74 (M = Mg, Mn, Fe, Co, Ni, Cu, Zn) under an external electric field is investigated. Simulation results suggest that an external electric field enhances the π* back-bonding between the transition metal and dinitrogen molecule while weakening the σ bond between the metal and other small gas molecules such as CO_2_ and CH_4_. In particular, Co-MOF-74 and Fe-MOF-74 show the highest dinitrogen binding energy in the presence of an electric field, twice as high as that of methane. Our work demonstrates that the asymmetric effect of the electric field on different gas molecules can serve as another dimension of design that can be exploited in small gas molecule separation in metal–organic frameworks.

## Abstract 2

External electric field increases the π* back-bonding (metal–N_2_) while weakening the σ bond (metal–CH_4_). This asymmetric effect enhances N_2_/CH_4_ separation performance in MOF-74.

## Graphical abstract

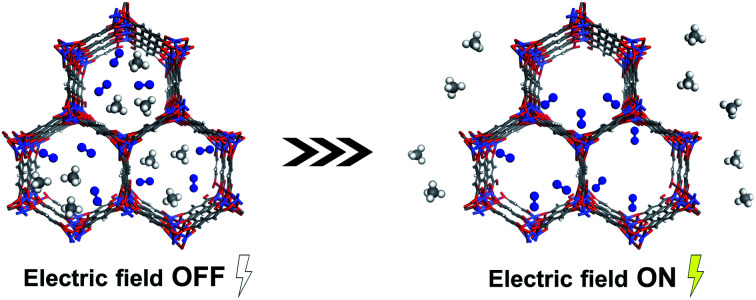

## Introduction

Dinitrogen, the most abundant species in the air, is widely known as a component that needs to be removed for utilization in raw mixtures such as natural gas. Cryogenic distillation is known to be useful for large-scale N_2_/CH_4_ separation application, but massive power consumption is one of the drawbacks.^1^ As such, alternative methods such as pressure swing adsorption and membrane separation have been explored to remedy these concerns.^2^ Recently, many researchers have used metal–organic frameworks (MOFs) to separate the mixture of N_2_ and CH_4_,^3–5^ and in particular, researchers have reported that the π* back-bonding between a MOF and dinitrogen can enhance the performance for the N_2_/CH_4_ separation.^6^

In terms of materials applications, external stimuli and modifications such as pressure, temperature, ligand insertion and electric/magnetic fields provide means to tune the properties of the materials,^7–9^ and thereby can be used as a “switch” to enhance the performance of the material. In particular, electric fields have been gaining traction as of late as their utility has been explored in many different applications such as gas separation of propene/propane^10^ and electric field-induced assembly of MOF.^11^ Moreover, there have been many computational studies regarding electric field that have demonstrated its potential utility in CO_2_ capture,^12^ methane C–H bond activation,^13^ graphene hydrogenation^14^ and controllable molecular gate.^15^ However, to the best of knowledge there hasn't been anyone who has investigated how electric field can affect the N_2_/CH_4_ separation performance.

Here, we use density functional theory (DFT) to investigate the mechanisms in which the electric field can asymmetrically modify both the N_2_ and the CH_4_ binding energies in M-MOF-74 (with M being Mg and 3d transition metals). We hypothesize that since the donation of electron in π* back-bonding is opposite to that of σ bond, there will be disparate effect on the bonds from the external electric field. And this difference will map into unique effects in the N_2_ and CH_4_ binding energies, which can lead to synergetic separation performances.

## Computational methods

**Fig. 1:**
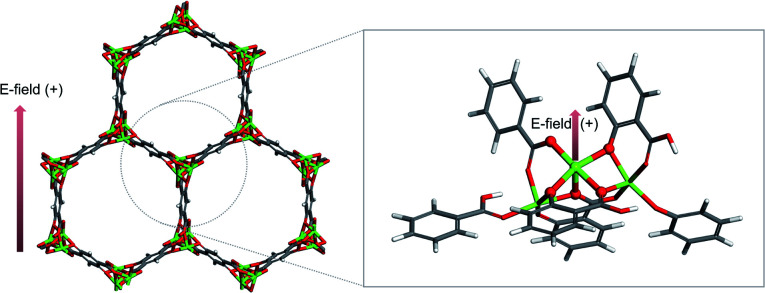
Cluster model description. From the periodic unit cell of Mg-MOF-74 (left), cluster model consists of 88 atoms is cleaved out (right). Direction of external electric field is indicated by the red arrow. Atoms are colored by its element type; green: magnesium, red: oxygen, grey: carbon, white: hydrogen.

The cluster model of M-MOF-74 is constructed in the same manner as the iron MOF-74 model from Verma *et al.*,^16^ that cleaves out the cluster by considering metal node as the center and drawing a circle to include linkers (see the circle in grey dashed line in Fig. 1 left). Periodic unit cell of Mg-MOF-74 from Lee *et al.*^17^ is used as a starting material for all the other M-MOF-74 structures. First, the Mg-MOF-74 unit cell is extended to cleave out the cluster model of 88 atoms that consists of three Mg atoms and six organic linkers (see Fig. 1).

When the cluster is cleaved out, functional groups such as carboxylate and oxido are terminated with hydrogen atoms to make the cluster neutral in charge. Then, to investigate the MOF-74 system with transition metals, the Mg atom is substituted with transition metal atoms such as Mn, Fe, Co, Ni, Cu, Zn which to the best of our knowledge are the list of experimentally synthesizable M-MOF-74s. Here we only substitute the central Mg atom to the other transition metals, so that the cluster can precisely simulate the environment of targeted M-MOF-74 while lowering computational cost. Spin state of the central transition metal is set to be a high-spin state (*S* = 5/2 for Mn, 4/2 for Fe, 3/2 for Co), which is deemed to be ground states, experimentally and computationally.^17–19^

All the DFT calculations in this study is performed using the Gaussian 16 program^20^ (G16), and M06-L^21^ exchange–correlation functional and def2tzvp^22^ basis sets, that are well validated as appropriate functional and basis sets for 3d transition metals by Xu *et al.*,^23^ are employed. During the geometrical optimization, the central metal atom and the first coordination shell composed of five oxygen atoms are relaxed and other atoms of the cluster are fixed. External electric field is applied to the cluster in the range of −0.010 a.u. to +0.010 a.u. (where 1 a.u. = 5.142 × 10^11^ V m^−1^). The positive direction of the electric field corresponds to the vector with direction as follows (see Fig. 1).

Binding energy of small gas molecules (CH_4_, CO_2_, N_2_) within the M-MOF-74 cluster is computed using the below equation.(Binding energy) = |*E*_cluster+gas_ − *E*_cluster_ − *E*_gas_|

Along with the binding energy, N_2_ stretching frequency, which is a good indicator of the π* back-bonding, is computed. Also, natural bond orbital (NBO) analysis is performed using the NBO 3.1 program^24^ (included in the G16). From the NBO analysis, stabilization energy *via* delocalizing donor NBO (Lewis type) to acceptor NBO (non-Lewis type) is calculated by second order perturbation theory as following
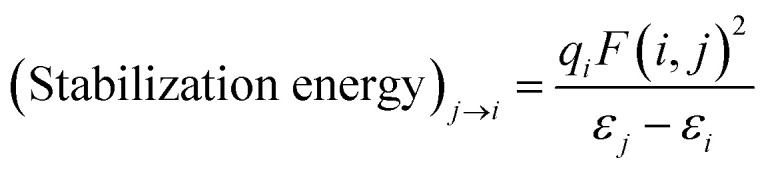
where *q*_*i*_ is occupancy of the donor NBO, *F*(*i*, *j*) is the off-diagonal NBO Fock matrix element, and *ε*_*i*_, *ε*_*j*_ are diagonal elements (orbital energies). The stabilization energy less than 0.5 kcal mol^−1^ is ignored. Visualization of NBO is carried out by GaussView 6.1.^25^

## Results and discussion

**Fig. 2:**
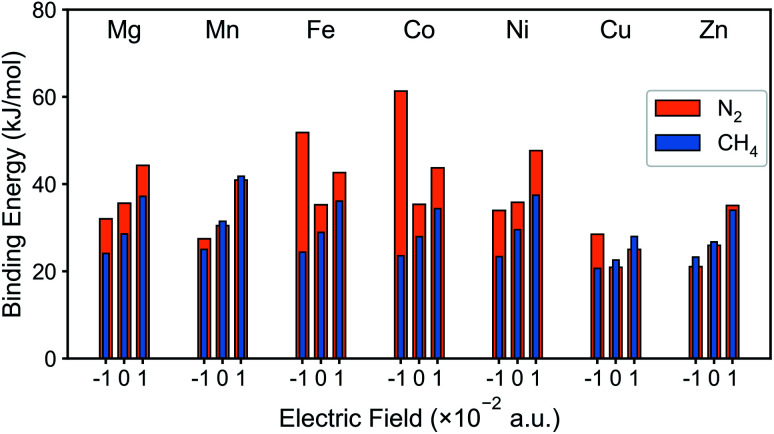
N_2_ and CH_4_ binding energy of M-MOF-74 (M = Mg, Mn, Fe, Co, Ni, Cu, Zn) with external electric field (−0.010 a.u., neutral, and +0.010 a.u.).

**Fig. 3:**
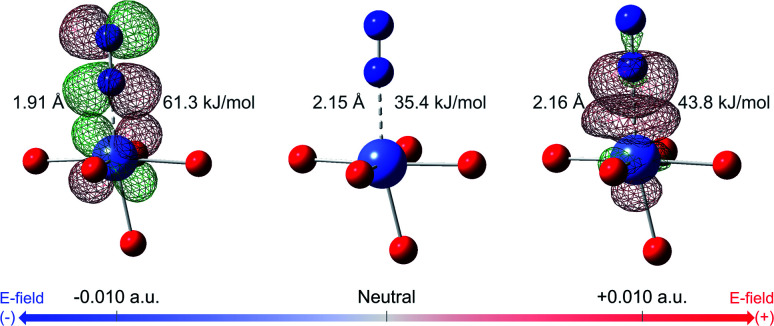
Visualization of NBOs in Co-MOF-74 and N_2_. NBOs corresponding to π* back-bonding, 3d orbital of Co (donor) and empty π* orbital of N_2_, are visualized at electric field of −0.010 a.u. NBOs corresponding to σ bond, lone pair electron of N_2_ and empty 3d orbital of Co, are visualized at electric field of +0.010 a.u. Binding energy of N_2_ and its bond length are written together. Color code: purple (cobalt), red (oxygen), blue (nitrogen), red mesh (positive surface), green mesh (negative surface).

The DFT binding energy of N_2_ and CH_4_ under electric field is computed for the M-MOF-74 structures and the results are plotted in Fig. 2 for different metal atoms. As the electric field changes from negative to zero to positive value, binding energy of CH_4_ shows monotonic increase for all metal types. Since σ bond is the dominant mechanism for CH_4_ binding in M-MOF-74, positive electric field further induces the electron to move from the CH_4_ gas molecule towards the coordinatively unsaturated metal site (CUS), thereby strengthening the σ bond. On the contrary, the negative electric field will provide a force that induces electrons away from the metal site and as such weaken the CH_4_–MOF interactions. On the other hand, N_2_ binding shows a different trend because of the π* back-bonding *via* d–π* interaction (3d orbital from transition metal and π* orbital from N_2_ molecule). For all metal types, positive electric field strengthens the N_2_ binding energy, similar to the case for CH_4_. On the other hand, negative electric field weakens N_2_ binding in Mg, Mn, Ni, and Zn-MOF-74, while it strengthens binding in Fe, Co, and Cu-MOF-74. Similar to CH_4_, the former can be interpreted as σ bond dominant environment, while for the latter case, π* back-bonding needs to be considered. In the M-MOF-74 systems, the π* back-bonding is formed by donating electrons from the d orbitals of the transition metal to the π* orbital of N_2_. Therefore, negative electric field induces the electrons to move toward the metal and thereby strengthening the π* back-bonding and increasing the N_2_ binding energy. Fig. 3 visualizes corresponding NBOs in Co-MOF-74, which showed the largest enhancement in the N_2_ binding energy for negative electric fields amongst the investigated systems. Similar analysis for CO_2_ can be seen in Fig. S1† where CO_2_ acts more like CH_4_ given that it also lacks the π* back-bonding.

**Fig. 4:**
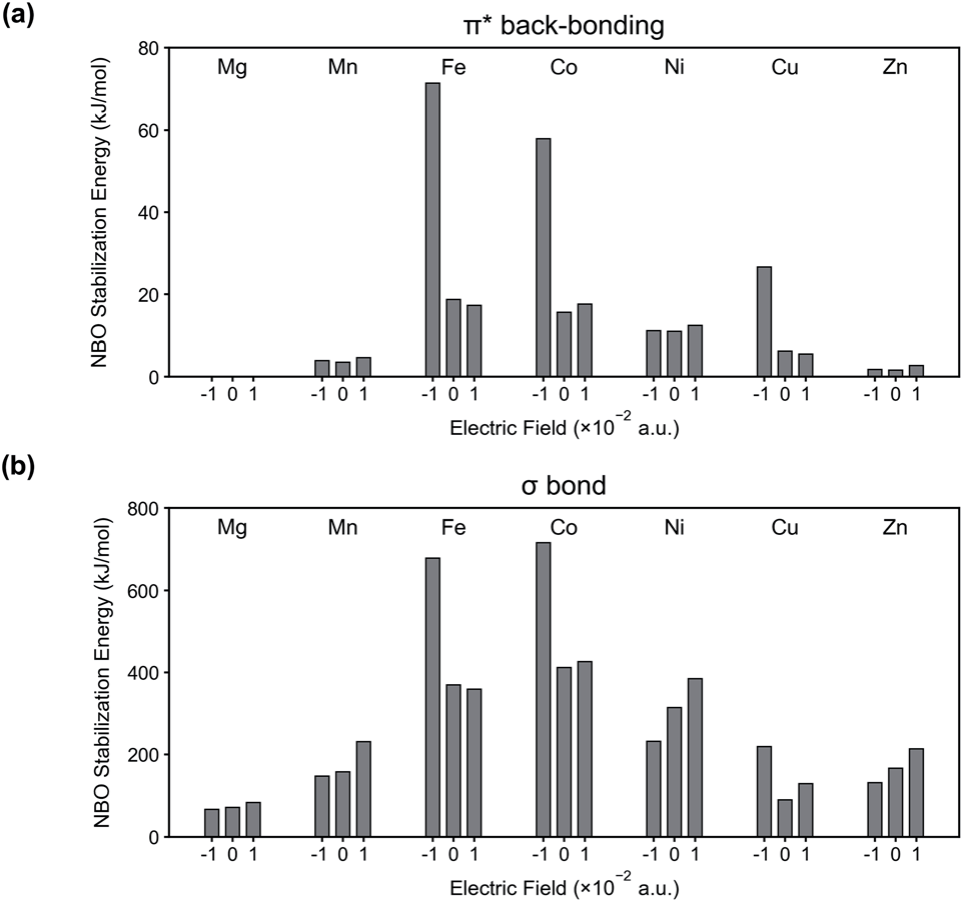
NBO stabilization energies corresponding to (a) π* back-bonding and (b) σ bond in the system of M-MOF-74 (M = Mg, Mn, Fe, Co, Ni, Cu, Zn) and N_2_.

To further validate our interpretation, NBO analysis is conducted from the binding energy calculations (see Fig. 4). In Fig. 4(a), stabilization energies corresponding to π* back-bonding are calculated and plotted. Similar to the previous interpretation, the stabilization energy increases in the system where the N_2_ binding energy is increased when an electric field is applied in the negative direction.

Along with π* back-bonding, the stabilization energies from σ bond (lone pair electrons (donor) of N atom in N_2_ → metal's antibonding lone pair orbitals (acceptor)) are computed as well (Fig. 4(b)). According to our previous assumption, we expect the amount of σ bond to increase for positive electric field and to decrease for negative electric field. The stabilization energy in Fig. 4(b) are well explained by this assumption except certain M-MOF-74 (*i.e.* M = Fe, Co, Cu) at negative electric field. The exceptions have significant increase of the π* back-bonding in common, and this strong π* back-bonding decreases the distance between N_2_ and CUS (see Fig. 4(a) for increased π* back-bonding and Table S1† for decreased bond length). Consequently, it increases overlap of orbitals participating in σ bond. Fig. S2† shows the increased overlap between orbitals participating in the σ bond and decreased bond length in Co-MOF-74 and N_2_ system. Therefore, a system with strong π* back-bonding shows synergetic increase for the σ bond at negative electric field. In Fig. S4 and S5,† the reason behind the superior performance of Fe, Co-MOF-74 is explained by considering the d orbitals splitting and the synergetic increase of the σ bond. In addition, NBO analysis for CH_4_ and CO_2_ with each M-MOF-74 systems are in ESI (see Fig. S6 and S7†).

**Fig. 5:**
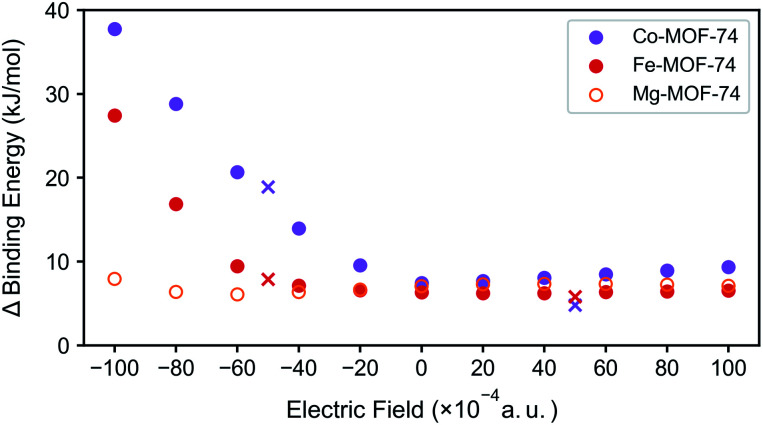
Binding energy difference between N_2_ and CH_4_ in M-MOF-74 (M = Co, Fe, Mg) under external electric field. Marker ‘×’ represents the rotated clusters' case.

For N_2_/CH_4_ separation, the DFT binding energies can serve as good predictors especially when comparing structures with the same topology in materials such as M-MOF-74 structures. As such, the difference between the N_2_ and CH_4_ binding energies for three test case M-MOF-74 systems (Mg-MOF-74, Co-MOF-74, and Fe-MOF-74) are plotted in Fig. 5 for several values of the electric fields. Mg-MOF-74 represents the MOF-74 system which showcases σ bond dominance, whereas Co-MOF-74 and Fe-MOF-74 are two systems that show the largest enhancement in the N_2_ binding energy at negative electric field. In Mg-MOF-74, the binding energy difference between N_2_ and CH_4_ remains relatively unchanged for different values of the electric field as both the N_2_ and the CH_4_ bind to CUS *via* σ bond, therefore cancelling out the effect of electric field. For Co-MOF-74 and Fe-MOF-74, similar trends can be found for positive electric field values. However, for negative electric fields, negative electric field increases the N_2_ binding energy while weakening CH_4_ binding energy because it strengthens the π* back-bonding and weakens the σ bond. Therefore, the binding energy difference between N_2_ and CH_4_ starts to increase as electric field becomes more negative. The increased N_2_ binding energy for negative electric field is accompanied by a decrease in N_2_ stretching frequency (see Fig. S3†), indicating the increase in the π* back-bonding.

In all of the aforementioned analysis, the direction of the electric field was purposefully chosen to optimally enhance the N_2_/CH_4_ separation. However, in real experiments, it is impossible to control the direction of the electric field with respect to the crystal orientation. As such, we explore the effect of the changes in the direction of the electric field while starting with the assumption that the changes in the binding energy will be equivalent to the component of the electric field that are aligned to the optimal direction. To test this assumption, the electric field is applied at the intensity of 0.01 a.u., but in the direction of the field is tilted to around 60 degrees. The resulting data points (Fig. 5, marked ‘×’) situated in the region that coincides between the inner product between electric field and the vector of unsaturated metal direction, validating our assumption.

## Conclusions

The effect of electric field as external stimuli to control the binding property of small gas molecules in MOF have been investigated computationally at the DFT level. Our computational studies indicate that due to the presence of π* back-bonding, certain M-MOF-74 systems show stronger binding for N_2_ for both positive and negative electric field directions whereas CH_4_ displays more of a monotonical behavior with respect to electric field. As such, one can theoretically use the electric field to increase the binding energy difference between N_2_ and CH_4_ in these systems, and thereby enhance the N_2_/CH_4_ separation performances. It remains to be seen how much of this work will be mapped to actual experimental studies given the large magnitudes of the electric field required to see meaningful effects from our computational studies. However, it has been shown in other computational/experimental work^10^ that electric fields require to see noticeable changes in macroscopic properties can be quite different and as such, it is our opinion that one cannot just dismiss the theoretical studies due to the large magnitude of electric field values.

## Conflicts of interest

There are no conflicts to declare.

## Supplementary Material

RA-012-D2RA04216A-s001
