# Supplementary material for: Theoretical study of induced selective N2 binding under an electric field in MOF-74: application for N2/CH4 separations
Source: RSC Adv. 2022 Aug 19;12(36):23396–9. doi: 10.1039/d2ra04216a (PMC9390010; doi:10.1039/d2ra04216a)
Supplement: RA-012-D2RA04216A-s001 [file RA-012-D2RA04216A-s001.pdf]

Electronic Supplementary Material (ESI) for RSC Advances.
This journal is © The Royal Society of Chemistry 2022

Supplementary Information

Theoretical Study of Induced Selective N_2 Binding under Electric Field in MOF-74: Application for N_2/CH_4 Separations

Honghui Kim and Jihan Kim*

Department of Chemical and Biomolecular Engineering, Korea Advanced Institute of Science and Technology (KAIST), 291 Daehak-ro, Yuseong-gu, Daejeon 34141, Republic of Korea

* Corresponding author: J.K. (jihankim@kaist.ac.kr)

M in M-MOF-74	E-field (a.u.)	Bond length of N_2-CUS(Å)	B.E.(N_2) (kJ/mol)	B.E.(CH_4) (kJ/mol)	B.E.(CO_2) (kJ/mol)
Mg	−0.010	2.42	32.1	24.1	40.7
	0	2.40	35.6	28.6	47.9
	+0.010	2.34	44.3	37.2	59.1
Mn	−0.010	2.59	27.5	25.1	33.1
	0	2.59	30.5	31.5	39.8
	+0.010	2.47	40.9	41.8	51.6
Fe	−0.010	1.99	51.9	24.4	32.5
	0	2.24	35.3	28.9	38.4
	+0.010	2.24	42.7	36.1	47.1
Co	−0.010	1.91	61.3	23.6	31.9
	0	2.15	35.4	27.9	38.3
	+0.010	2.16	43.8	34.4	46.4
Ni	−0.010	2.23	34.0	23.4	31.9
	0	2.21	35.8	29.6	43.9
	+0.010	2.15	47.7	37.5	54.1
Cu	−0.010	2.19	28.5	20.7	23.8
	0	2.53	21.0	22.6	27.6
	+0.010	2.46	25.0	28.0	33.0
Zn	−0.010	2.63	21.1	23.3	32.8
	0	2.55	26.0	26.8	36.7
	+0.010	2.41	35.1	34.0	43.3

Table S1. Binding energy of N_2, CH_4, CO_2 and bond length of N_2-CUS in M-MOF-74 (M = Mg, Mn, Fe, Co, Ni, Cu, Zn)

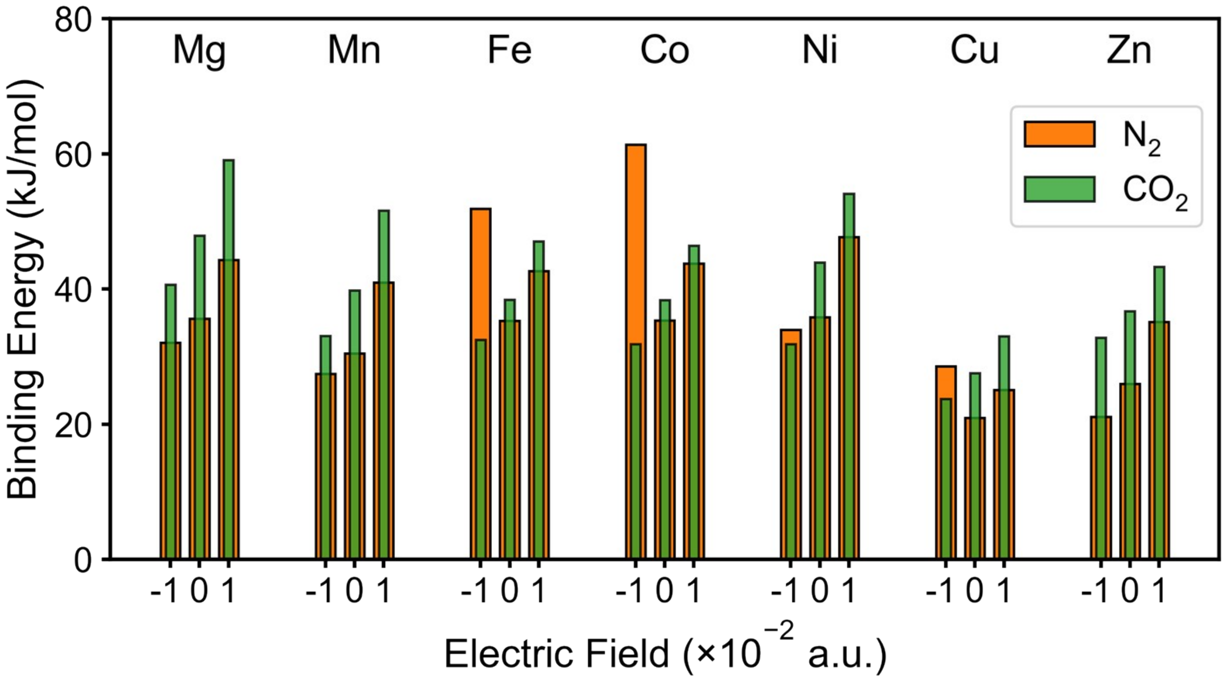

Figure S1. Binding energy of N_2 and CO_2 in the system of M -MOF-74 (M=Mg, Mn, Fe, Co, Ni, Cu, Zn).

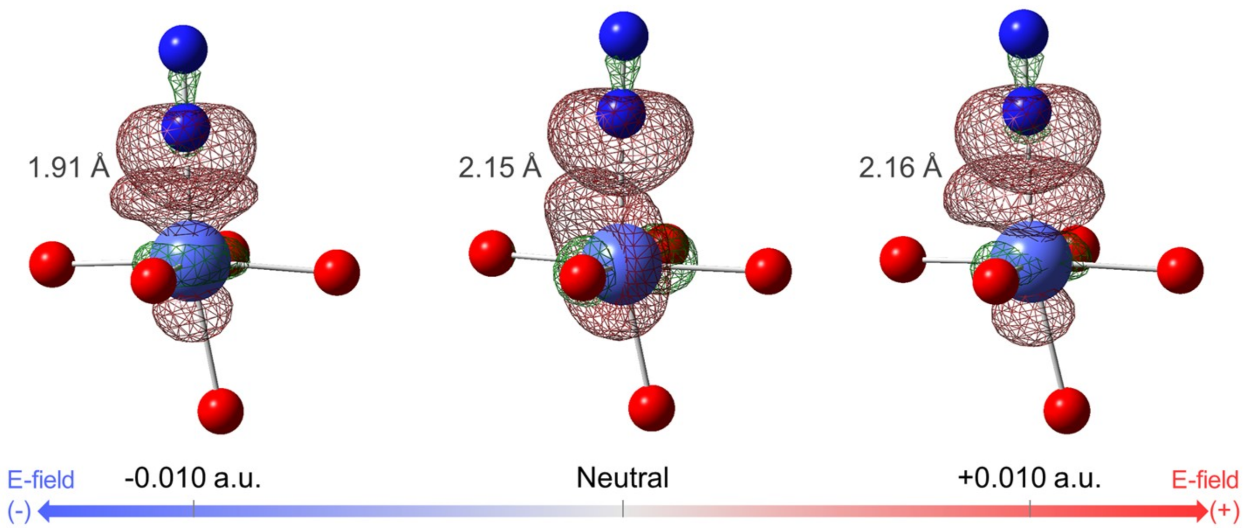

Figure S2. Visualization of NBOs corresponding to the σ bond (lone pair electrons (donor) of N atom in N_2 → metal's empty 3d orbitals (acceptor)) between N_2 and Co atom in Co-MOF-74 at negative, neutral, positive electric field. NBOs with largest stabilization energy for each case is visualized. Bond length between N_2 and Co atom is written together. Color code: Purple (Cobalt), Red (Oxygen), Blue (Nitrogen), Red mesh (positive surface), Green mesh (negative surface).

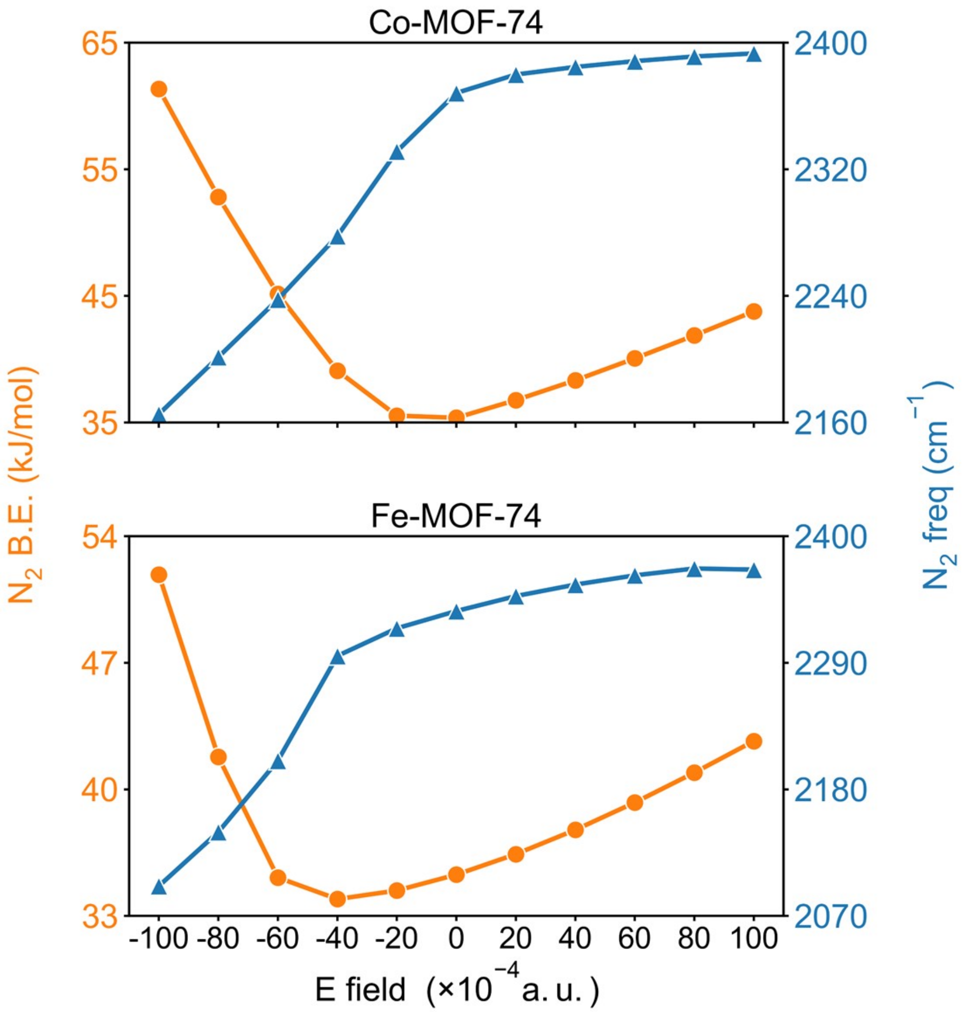

Figure S3. N_2 binding energy (orange circle) and its stretching frequency (blue triangle) in M-MOF-74 (M=Co, Fe) under external electric field.

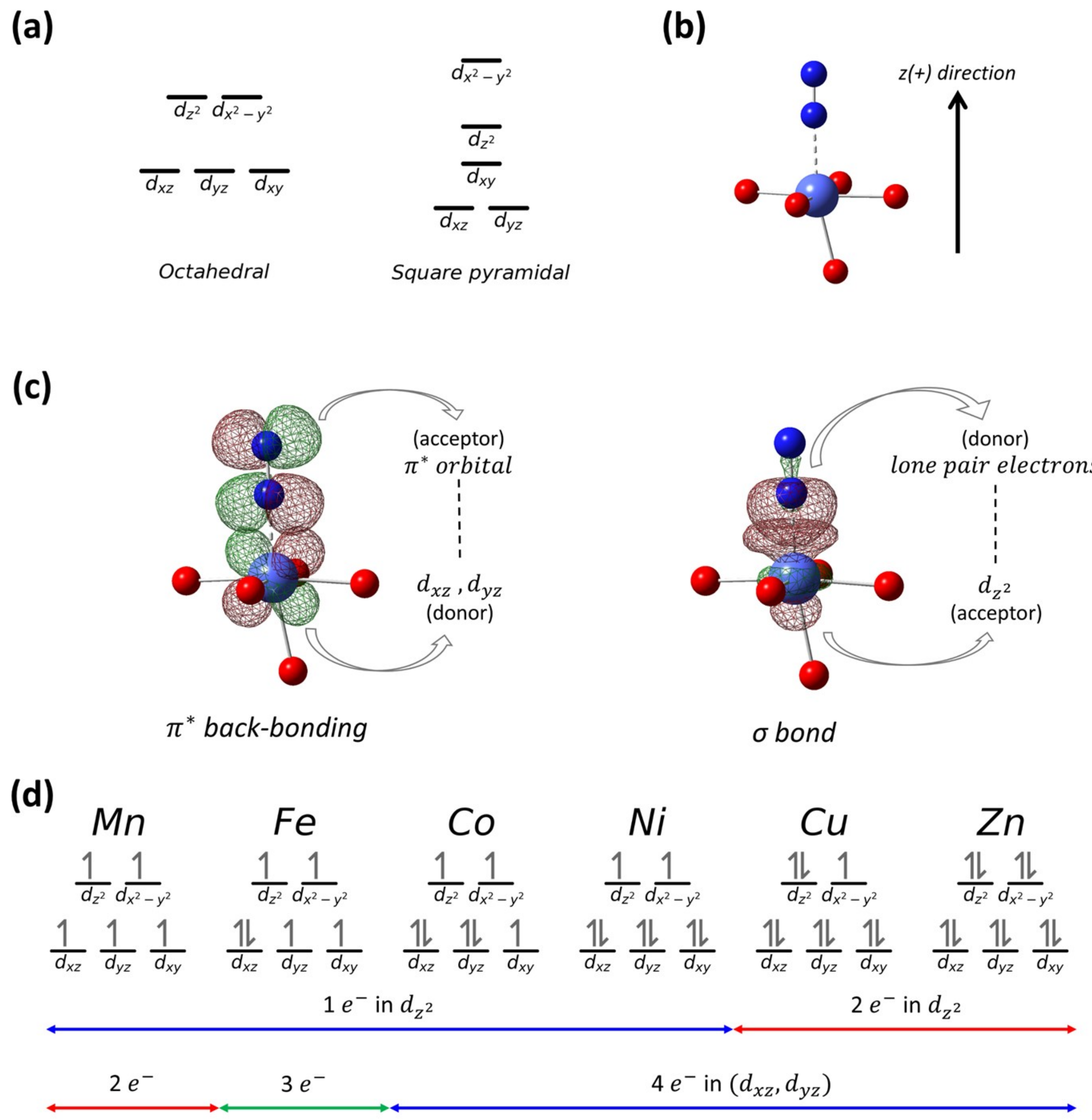

Figure S4. (a) Energy diagram of d orbitals in octahedral coordination geometry and square pyramidal. (b) Coordination geometry of metal center in M-MOF-74. Purple: metal atom, blue: Nitrogen, red: Oxygen. (c) Description of orbital interaction in π* back-bonding and σ bond. (d) Metal's d electron configuration of M-MOF-74 system (M=Mn, Fe, Co, Ni, Cu, Zn) and number of electrons in specific d orbitals (d_{z^2} and d_{xz}, d_{yz}).

Figure S4 shows coordination environment of metal center in M-MOF-74 and electron configuration of d electrons. Here we explain three key points to understand the extraordinary behavior of certain metals like Fe and Co.

Key 1: d electrons will be filled in the order of (d_{xz}, d_{yz})→d_{xy}→d_{z^2}→$d_{x^2-y^2}$.
Coordination geometry of the metal center in M-MOF-74 can be considered as octahedral or

square pyramidal. In Figure S4 (b), CUS forms octahedral coordination with N_2 molecule, but its energy state cannot be perfect octahedral coordination (e.g. Figure S4 (a) left). Because the bond between the metal and oxygen atom (in Figure S4 (b)) will be tighter than the bond between the metal and N_2 molecule, there will be less electron density interruption toward z-direction. Therefore, the energy state of d orbitals along the z-direction will be more stable, and d electrons will be filled in the order of (d_{xz}, d_{yz})→d_{xy}→d_{z^2}→$d_{x^2-y^2}$ similar to square pyramidal coordination. From this order, d orbitals in M-MOF-74in high spin state will be filled like Figure S4 (d).

Key 2: The more electron in (d_{xz}, d_{yz}), the stronger the $\pi*$ back-bonding.

Key 3: The less electron in d_{z^2}, the stronger the σ bond.

The description of which orbitals are involved in each bond ($\pi*$ back-bonding and σ bond) is in Figure S4 (c). In the case of $\pi*$ back-bonding, (d_{xz}, d_{yz}) orbitals act as donor. The more electron in (d_{xz}, d_{yz}), the stronger the $\pi*$ back-bonding. For stronger σ bond, it requires empty d_{z^2} because spare electron in d_{z^2} will be located in antibonding state when N_2 molecule donates its lone pair electrons to the metal. All things considered, more electrons in (d_{xz}, d_{yz}) orbitals and less electrons in d_{z^2} orbital are required to have strong N_2 binding.

Figure S4 (d) shows electron configurations in d orbitals for each M-MOF-74 and which of them satisfy the two keys (Key 2 and 3) to have strong N_2 binding. Co and Ni satisfy two keys the most and Fe also satisfies them moderately. In Figure 2, Co-MOF-74 shows better performance enhancement under electric field compared to Fe-MOF-74. Another interesting point is that these promising candidates (Co, Ni, and Fe-MOF-74) have higher carbon monoxide adsorption capacity among M-MOF-74 systems.[1] (Since carbon monoxide can also form $\pi*$ back-bonding with the metal atom, it has similarity with N_2 molecule.)

However, Ni-MOF-74 doesn't show such strong binding with N_2 under electric field. It appears that increased occupancy in d_{z^2} orbital causes the weak N_2 binding (see Figure S5). Under negative electric field, d_{z^2} occupancy of Ni atom in Ni-MOF-74 increased. The increased d_{z^2} occupancy (i.e. increased electron density at antibonding state) interrupt strong σ bond, and it will result in weak N_2 binding with Ni atom. Slightly decreased d_{z^2} occupancy of Cu under negative electric field appears to be related with increased N_2 binding in Figure 2.

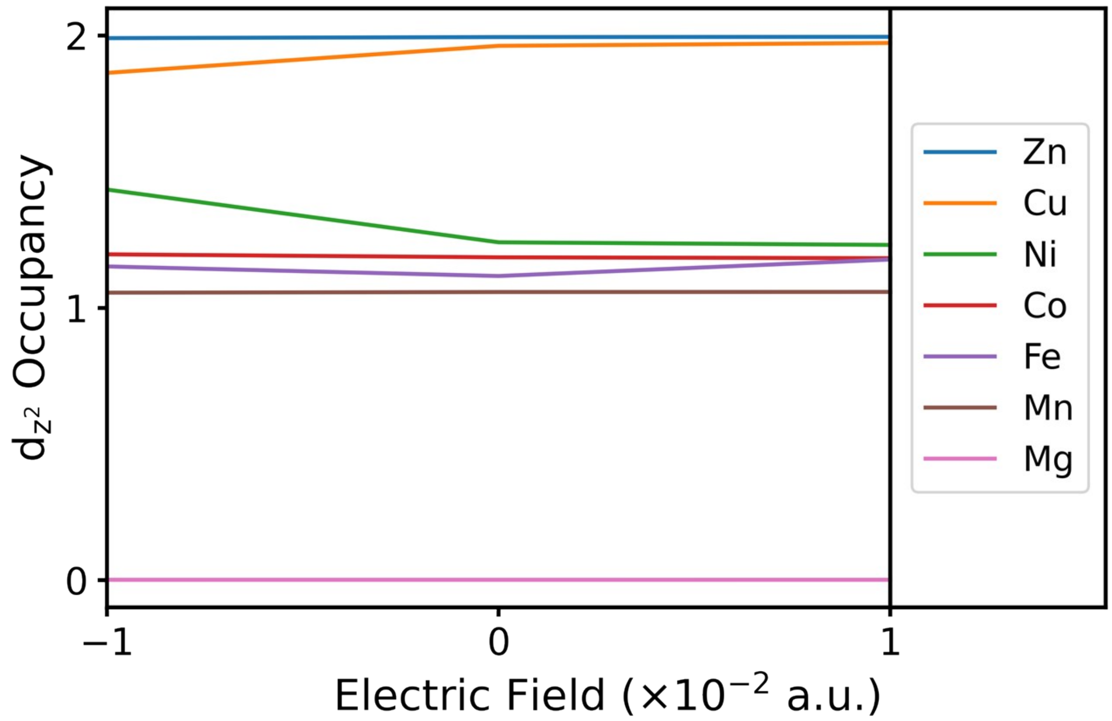

Figure S5. d_{z^2} occupancy of metal center in M-MOF-74 from natural population analysis.

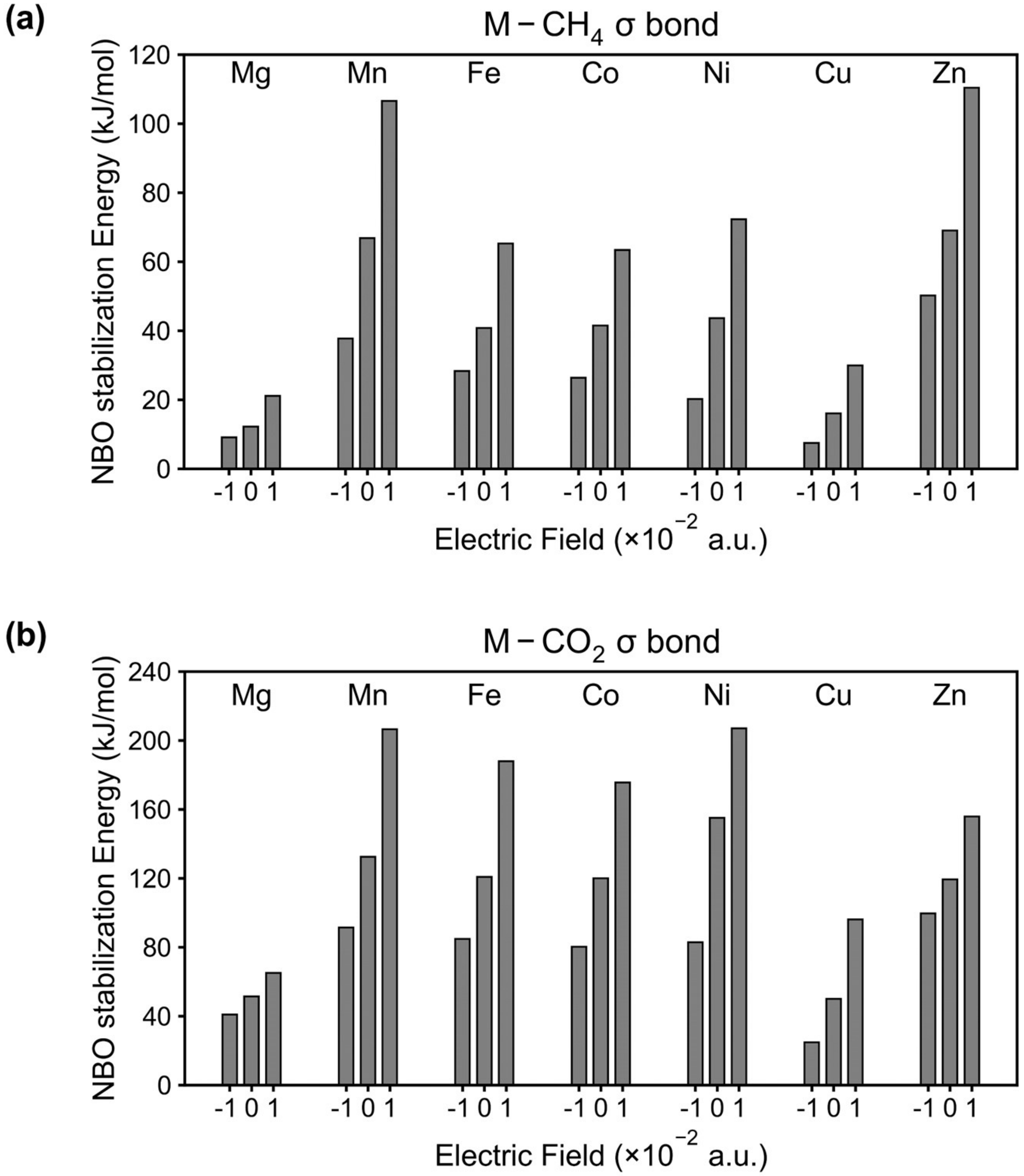

Figure S6. NBO analysis for **(a)** σ bond between CH_4 and CUS (C-H bond orbital in CH_4 (donor) → metal's antibonding lone pair orbital (acceptor)), and for **(b)** σ bond between CO_2 and CUS (O lone pair orbital in CO_2 (donor) → metal's antibonding lone pair orbital (acceptor))

Figure S6 shows NBO stabilization energy plots regarding to the σ bond between the transition metal and small gas molecules (CH_4 and CO_2). In both plots, the stabilization energy monotonically increases regardless to the metal type, and such result agrees with our hypothesis that these small gas molecules has σ bond dominance.

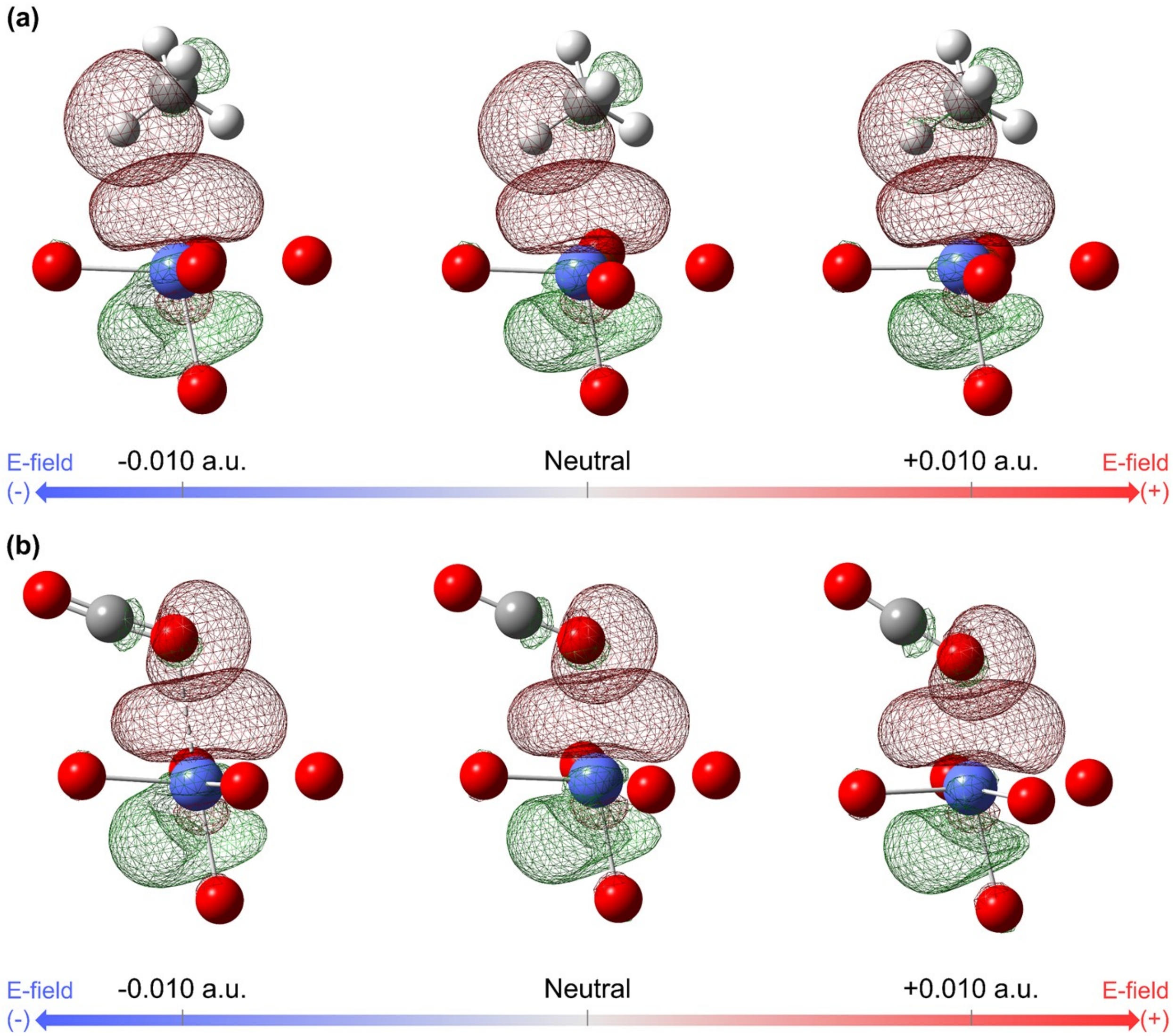

Figure S7. Visualization of NBOs corresponding to σ bond of **(a)** Co-CH_4 and **(b)** Co-CO_2 at negative, neutral, positive electric field in Co-MOF-74 system. Central transition metal of the cluster and its first coordination shell (5 oxygen atoms) are only visualized for simplicity. Color code: Purple (Cobalt), Red (Oxygen), Blue (Nitrogen), Red mesh (positive surface), Green mesh (negative surface).

Reference

1. E. D. Bloch, M. R. Hudson, J. A. Mason, S. Chavan, V. Crocellà, J. D. Howe, K. Lee, A. L. Dzubak, W. L. Queen, J. M. Zadrozny, S. J. Geier, L.-C. Lin, L. Gagliardi, B. Smit, J. B. Neaton, S. Bordiga, C. M. Brown and J. R. Long, *Journal of the American Chemical Society*, 2014, **136**, 10752-10761.
